# Melanoma-Derived Extracellular Vesicles Bear the Potential for the Induction of Antigen-Specific Tolerance

**DOI:** 10.3390/cells8070665

**Published:** 2019-07-02

**Authors:** Markus Düchler, Liliana Czernek, Lukasz Peczek, Wojciech Cypryk, Malgorzata Sztiller-Sikorska, Malgorzata Czyz

**Affiliations:** 1 Department of Bioorganic Chemistry, Centre of Molecular and Macromolecular Studies, Polish Academy of Sciences, 112 Sienkiewicza Street, 90-363 Lodz, Poland; 2 Department of Molecular Biology of Cancer, Medical University of Lodz, 6/8 Mazowiecka Street, 92-215 Lodz, Poland

**Keywords:** exosomes, extracellular vesicles, cancer immunosuppression, major histocompatibility complex, MHC-I transfer, IL-6, TGF-beta, melanoma

## Abstract

Background: Cancer-induced immunosuppression is antigen-specific rather than systemic and the mechanisms for the antigen specificity are incompletely understood. Here we explore the option that tumor-associated antigens (TAAs) may be transferred to antigen-presenting cells (APCs), together with immunosuppressive molecules, through cancer-derived small extracellular vesicles (sEVs), such as exosomes. Stimulation of a suppressive phenotype in the very same APCs that take up TAAs may yield antigen-specific tolerance. Methods: sEVs isolated from patient-derived or well-established melanoma cell lines were used to demonstrate the transfer of major histocompatibility complex (MHC) molecules to the surface of APCs. The immunosuppressive influence of sEVs was assessed by flow cytometry analysis of activation markers, cytokine expression, and mixed lymphocyte reactions. Results: MHC class I molecules were transferred from melanoma cells to the cell surface of APCs by sEVs. Concomitantly, CD86 and CD40 co-stimulatory molecules were down-regulated and IL-6 production was strongly induced. TGF-β transported by sEVs contributed to the promotion of a suppressive phenotype of APCs. Conclusion: The presented results indicate the existence of a hitherto undescribed mechanism that offers an explanation for antigen-specific tolerance induction mediated by cancer-derived sEVs.

## 1. Introduction

Melanoma belongs to the group of tumors that are highly sensitive to immunotherapies. The therapeutic antibodies targeting the immune checkpoints, ipilimumab for cytotoxic T-lymphocyte antigen-4 (CTLA-4) and nivolumab and pembrolizumab for programmed death 1 (PD-1), greatly increased the average life expectancy for patients with metastatic melanoma. However, about half of all patients demonstrate primary or acquired resistance [1,2]. A better understanding how melanoma cells modulate the immune system is clearly needed. Here, we provide evidence that melanoma-derived sEVs contribute to antigen-specific immunosuppression.

Cancer development can be suppressed by the host immune system [3]. Cytotoxic T cells constitute a part of the adaptive immune response that very efficiently kills cancerous cells. The activation of cytotoxic T cells depends on their direct interactions with antigen presenting cells (APCs) which take up tumor-associated antigens (TAA), process them into peptides, and present them on MHC (major histocompatibility complex) molecules [4,5]. In the immune synapse, the contact zone between APCs and T cells, the interaction of MHC molecules with the cognate T cell receptor (TCR) ensures the specificity to a given antigen [6]. Whether TCR engagement results in activation or suppression of T cells depends on local availability of additional co-stimulatory or inhibitory signals. The co-stimulatory signals required for the differentiation and proliferation of T cells include the interaction of the CD28 co-receptor on T cells with CD80 and/or CD86 on APCs and cytokine signaling, e.g., by interleukin 12 (IL-12) [7,8]. IL-12 production by APCs is stimulated by CD40 binding to its ligand (CD40L, CD154) on T helper cells that participate in the activation of cytotoxic T cells when they recognize TAA peptides presented by MHC II molecules on the APC surface. The integration of multiple signals for T cell stimulation comprises a safety mechanism preventing undesirable and dangerous activation of cytotoxic T cells. 

In addition, T cell activation is negatively regulated by strong inhibitory mechanisms. Binding of MHC molecules to TCRs in the absence of co-stimulation results in T cell anergy [9,10]. T cells can express inhibitory receptors, such as CTLA-4 and PD-1, whose interaction with CD80 or PD-L1, respectively, prevents T cell activation [11,12]. Interruption of these inhibitory signals is recently broadly exploited in the development of immunotherapy against melanoma and other kinds of cancer [1,13]. T cell activation requires fully maturated APCs, as immature or semi-mature DCs are tolerogenic, converting naïve T cells into regulatory T (Treg) cells, which are potent antigen-specific suppressors of immune reactions able to keep cytotoxic T cells inactive [9,10]. 

The establishment of a tumor requires the escape from immune surveillance. Some efficient strategies implemented by cancer cells to interfere with antigen presentation and activation of a cytotoxic T cell response includes the prevention of DC maturation, down-regulation of MHC molecules and the co-stimulatory receptors, inhibition of cytokine production, and induction of inhibitory receptor expression [14]. These effects are induced by receptor-ligand interactions and the secretion of immunosuppressive mediators, such as IL-10, TGF-β, VEGF, or prostaglandin E2 (PgE2) [15,16,17]. Interestingly, the establishment of a tumor in the host organism includes the suppression of cytotoxic T cell reactions in an antigen-specific way; not T cell reactivity in general, but only the specific immune reactions to tumor-associated antigens (TAA) are suppressed [18]. The mechanisms enabling such antigen-specific immune suppression are not entirely understood.

Apart from direct cell-cell contacts and secreted soluble factors, tumor cells also use exosomes for intercellular communication [19,20]. Exosomes are 30–150 nm sized vesicles generated in multivesicular bodies, a late endosomal compartment. Exosomes are released into the extracellular environment and can be taken up by other cells to function in intercellular communication through the exchange of proteins, lipids, functional mRNAs, and microRNAs (miRNAs) that can reprogram the gene expression in recipient cells at the post-transcriptional level [21]. Cancer-derived exosomes are capable of either stimulating or suppressing immune reactions [22,23,24]. For example, by transferring TAA to APCs or direct stimulation of T cells through MHC molecules within their membrane, exosomes exert immune stimulatory activity by transferring suppressive cytokines or ligands for inhibitory receptors. Exosomes prohibit anti-cancer immunity [25]. Importantly, exosomes can transport functional MHC class I and class II molecules with bound TAA-derived peptides, a process that is called cross-dressing [26]. We hypothesized that the TAA peptide-MHC complexes carried by sEVs might participate in the antigen-specific tolerance induction. According to this hypothesis, the vesicles transfer TAA-derived peptide-MHC complexes together with immunosuppressive cytokines and miRNAs to APCs. The TAA-MHC complexes would confer the antigen specificity, while the co-transferred cytokines and nucleic acids would concurrently modulate gene expression in APCs to suppress immune responses and induce tolerance. 

As the separation of exosomes from other extracellular vesicles with the same density is not achievable with the isolation techniques applied, we use the term ‘small extracellular vesicles’ (sEVs) instead of exosomes throughout the study, although the characterization of the isolated sEVs suggest that exosomes constitute the majority of our particle preparations.

## 2. Materials and Methods

### 2.1. Cell Culture

#### 2.1.1. Antigen-Presenting Cells (APCs) 

The human monocytic leukemia cell line THP-1 (kindly provided by Prof. J. Dziadek, Polish Academy of Sciences, Lodz, Poland) was cultured at 5 × 10^5^–1 × 10^6^ cells/mL in RPMI 1640 medium, supplemented with 10% FBS, 2 mM l-glutamine, 0.168 mM penicillin, 0.172mM streptomycin, 0.05 mM β-mercaptoethanol, and 1mM sodium pyruvate in a humidified incubator at 37 °C and 5% CO_2_ (Binder GmbH, Tuttlingen, Germany). Primary human monocytes were isolated from the blood of healthy donors by a magnetic cell separation procedure using anti-CD14 antibodies (MACS system, Miltenyi Biotech, Bergisch Gladbach, Germany). The MACS system was also used to isolate T cells with the pan T cell isolation kit (MACS system, Miltenyi Biotech). The differentiation of primary monocytes or THP-1 cells into dendritic cells (DCs) was achieved by culturing the cells in the presence of recombinant human IL-4 (100 ng/mL) and GM-CSF (100 ng/mL) for three to five days. For the stimulation of cytokine production, cells were incubated at a density of 5 × 10^5^ cells per mL in serum-free medium (AIM V, Gibco–Thermo Fisher Scientific, Life Technologies Polska, Warsaw, Poland) for 24 h with LPS (Lipopolysaccharide (E. coli 0111:B4), 1 µg/mL; Sigma-Aldrich, St Louis, MO, USA) and CD40 ligand (100 ng/mL, soluble, human, recombinant Mega CD40L, Enzo Life Sciences, Lausen, Switzerland). 

#### 2.1.2. Melanoma Cell Culture for sEV Production

The human melanoma cell line A375 (a gift from Prof. Piotr Laidler, Jagiellonian University, Krakow, Poland) was maintained in RPMI 1640 medium supplemented with 10% FBS and antibiotics as before. For the elimination of bovine serum derived sEVs, growth media were subjected to ultracentrifugation at 100,000× *g* for 2.5 h at 10°C (full acceleration and maximal breaking). For sEV production, cells were grown in this sEV-depleted medium. Melanoma cell lines from drug-naïve patients were also used as source of sEVs. For that, an approval by Ethical Commission of Medical University of Lodz was obtained and patients consented to tissue acquisition. Patient-derived melanoma cell lines, DMBC12 and DMBC21, were grown in non-adherent flasks in stem cell medium as described [27].

### 2.2. Isolation and Characterization of Extracellular Vesicles 

Tumor cell-derived sEVs were isolated by differential centrifugation [28]. After the cell cultures reached high density (about 90% confluency for adherent cells, about 10^6^ cells/mL for suspension cells), cells were pelleted by centrifugation at 300× *g* for 4 min. The remaining supernatants were centrifuged for 30 min at 10,000× *g* (10 °C) to precipitate cell debris and organelles, then sEVs were pelleted by ultracentrifugation at 100,000× *g* for 2.5 h (10 °C, full acceleration and breaking power). The sEV pellet was re-suspended in 11 mL phosphate-buffered saline (PBS) and the last centrifugation step was repeated. The pellet was again re-suspended in PBS and the protein concentration was determined by the Bradford assay (Bio-Rad Polska, Warsaw, Poland). The identity of sEVs was confirmed through the detection of characteristic surface markers [28]. EVs were adsorbed to latex beads, stained with anti-CD63 or anti-CD9 antibodies, and analyzed by flow cytometry. Staining with not-specific isotypic antibodies served as a negative control. Melanoma-derived sEVs (A375) in a concentration of 0.1 µg/µL were further analyzed by atomic force microscopy using an NTEGRA SPECTRA instrument (NT-MDT, Spectrum Instruments Ltd., Limerick, Ireland) [29]. For the visualization of EVs by Transmission Electron Microscopy (TEM, Tesla BS 512 with YAG camera, Brno, Czech Republic) the vesicles were fixed with 2% paraformaldehyde. The sEV suspension was loaded onto formvar carbon coated electron microscopy grids (FCF200-Cu-50, 200 mesh, Electron Microscopy Sciences, Hatfield, PA, USA), fixed in 1% (*v*/*v*) glutaraldehyde, and contrasted with 2% (*w*/*v*) aqueous uranyl acetate (Polysciences) [30].

### 2.3. Flow Cytometry

Flow cytometry measurements were performed on a FACSCalibur (Becton-Dickinson, East Rutherford, NJ, USA). For each sample, a minimum of 10^4^ cells or beads was measured. Results were analyzed using the CellQuest software (Becton-Dickinson). On myeloid cells, the surface markers CD40, CD80, CD86, HLA-DR, and HLA-ABC were assessed. Antibodies labeled with fluorescein (HLA-DR), phycoerythrin (CD80, HLA-ABC), or apocyanin (CD86, CD40) were purchased from Biolegend (San Diego, CA, USA) and Becton-Dickinson. For the flow cytometric analysis of sEVs, the vesicles were adsorbed to aldehyde-sulfate latex beads (3.8 µm size, Life Technologies Polska, Warsaw) for 20 min at room temperature. BSA was used to saturate the binding sites of the beads, followed by incubation with glycine (100 mM) in PBS for 20 min at room temperature. The beads were washed twice with FACS buffer (PBS, 0.5% BSA, 0.1% Na-azide) incubated with FITC-conjugated or PE-conjugated monoclonal antibodies for 1h in the dark, washed again with cold FACS buffer, and analyzed. Cytokines (IL-12, IL-6, VEGF, TGF-β) from cell culture supernatants were quantified using cytometric bead arrays (Becton-Dickinson), according to the accompanying instructions. 

### 2.4. Intercellular Transfer of Peptide-MHC-I Complexes

To demonstrate the transfer of MHC class I molecules to the cell surface of APCs, fluorescently labeled antibodies recognizing HLA-ABC were bound to melanoma sEVs in PBS (30 min at 0 °C). The mixture was diluted 10-fold and insoluble antibody aggregates were removed by centrifugation at 12,000× *g* for 10 min. The supernatant was further diluted (10-fold) and sEVs were pelleted by ultracentrifugation at 100,000× *g* for 2.5 h. The sEV pellet was re-suspended in cell culture medium and added to APCs at a concentration of 10 μg/mL. The transported fluorescence was measured 16 h later by flow cytometry. APC-labeled goat anti-mouse secondary antibodies (Biolegend) were used to demonstrate the appearance of the MHC-antibody complexes at the cell surface.

### 2.5. RNA Isolation and Real-Time Quantitative Reverse Transcription PCR (qRT-PCR)

Total RNA was isolated according to standard protocols using the TriPure Isolation Reagent, (Roche Diagnostics, Mannheim, Germany). The mRNA levels of CD40, CD80, CD86, HLA-A, ALA-B, HLA-C, and HLA-DRalpha were evaluated by real-time quantitative reverse transcription-polymerase chain reaction (qRT-PCR) using the LC RNA amplification kit SYBR Green I and a LightCycler Instrument 1.0 (Roche Diagnostics). The target gene expression levels were related to the house-keeping reference gene GADPH and to 5S ribosomal RNA. All primers used are listed in the supplementary Table S1. Products of amplification were identified by the thermal dissociation method.

### 2.6. Western Blot Analysis 

A whole cell extract from the A375 cell line was prepared by lysing ~ 3 × 10^6^ cells (confluent 25 cm^2^ culture flask,) in 0.3 mL RIPA Buffer (150 mM NaCl, 50 mM Tris-HCl pH 7.4; 0.1% Triton X-100, 2 mM EDTA, 0.1% SDS) containing a cocktail of protease inhibitors (Sigma Aldrich) on ice for 30 min. The lysate was centrifuged at 20,000× *g* for 15 min at 4 °C and the supernatant was collected. A Bradford assay was used to measure the protein concentration. Exosomes were lysed in Lammeli loading buffer by heating to 95 °C for 10 min. A total of 40 µg of protein per sample was separated on 8% SDS-polyacrylamide gels under denaturing conditions. A size marker (PageRuler™ Plus Prestained Protein Ladder, m.w. 10–250 kDa, Thermo Scientific) was included. After the electrophoresis, the proteins were transferred onto nitrocellulose membranes (Immobilon-P, Millipore), which were blocked in TBS-Tween solution (1× TBS + 1% Tween-20), containing 3% BSA, for 1.5 h at room temperature and incubated overnight at 4 °C with the primary antibodies (anti-human Alix, mouse monoclonal antibody, Santa Cruz Biotechnology, sc-271975, 1:500, Dallas, Texas, USA), and anti-human Calnexin (rabbit policlonal antibody, Sigma-Aldrich, C4731, 1:500). The following day, membranes were washed in TBS-Tween buffer three times for 15 min and incubated with the secondary antibodies labeled with horseradish peroxidase for 1.5 h at room temperature, (goat-anti mouse IgG-HRP: 926-80010, LI-COR, 1:10 000; goat-anti rabbit IgG-HRP, DAKO, P044801-2, 1:10 000). After washing the membranes in the TBS-Tween solution, bands were detected using enhanced chemiluminescence (ECL) (Westar Supernowa, CYANAGEN, XLS3 FS) and a G-Box system for analysis and documentation (Syngene).

### 2.7. Allogeneic Mixed Lymphocyte Reaction

PBMCs were isolated from the blood of healthy donors by Ficoll cushion centrifugation. PBMCs from one donor were re-suspended in cell culture medium (RPMI 1640 supplemented with 10% FBS, 2 mM l-glutamine, 100 IU/mL penicillin, 100 µg/mL streptomycin). Cell density was adjusted to 1 × 10^6^ cells/mL. PBMCs of a second donor were re-suspended in PBS and labeled with 5 µM CFSE (carboxyfluorescein diacetate succinimidyl-ester, Molecular Probes, InVitrogen) for 10 min at 37 °C. Labeled cells were washed four times with cell culture medium and suspended at 1 × 10^6^ cells/mL. A total number of 2 × 10^5^ labeled cells were mixed with the same amount of unlabeled cells from the first donor. The test was set up in triplicates (three independent experiments). The sEVs isolated from A375 melanoma cells were added at a concentration of 20 µg/mL. Maximal stimulation of T cell proliferation was achieved using PMA (Phorbol-12-myristate-13-acetate, 10 ng/mL) and ionomycin (500 ng/mL) in cultures containing only cells from the second donor. After 72 h, cells were harvested, stained with APC-labelled anti-CD3 antibodies and analyzed by flow cytometry. 

### 2.8. Statistical Analysis

Results from at least three independent experiments are presented as mean ± SD. Data were analyzed using Student’s paired *t*-test after confirming normal distribution by the Shapiro–Wilk test and a *p*-value below 0.05 was considered significant. 

## 3. Results

### 3.1. Isolation and Characterization of Melanoma Cell-Derived sEVs

**Figure 1:**
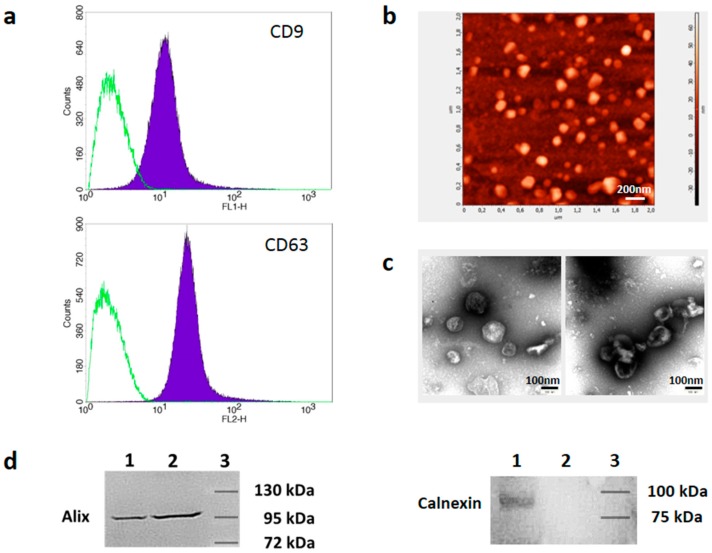
Characterization of melanoma cell-derived sEVs. (**a**) sEVs were adsorbed to latex beads, stained with anti-CD9-FITC and anti-CD63-PE antibodies, and analyzed by flow cytometry (filled peaks). Isotype control antibodies (open peaks) were used to demonstrate the specificity of staining (representative of n = 4). (**b**) Atomic force microscopy image of melanoma cell-derived sEVs. Scale bar, 200nm. (**c**) Images of melanoma-derived sEVs were obtained by TEM (transmission electron microscopy). Scale bar, 100nm. (**d**) Western blot analysis of cellular lysates (lane 1) and sEV lysates (lane 2) using antibodies binding to Alix or Calnexin, as indicated. Lane 3 shows the position of the marker bands. The molecular weight is indicated.

Melanoma cell lines were grown in serum-free (DMBC cell lines) or exosome-depleted medium (A375) and sEVs were isolated by differential centrifugation. About 3 × 10^7^ particles per µg of protein were obtained. The efficiency of removal of sEVs from the culture medium was confirmed by demonstrating decreased RNA concentration in the depleted medium to a level lower than in commercially available exosome-depleted media (not shown). The sEVs were characterized by detection of CD9 and CD63 proteins at their surface by flow cytometry after adsorption of the vesicles to latex beads (Figure 1a). Atomic force microscopy (AFM) analysis (Figure 1b) and transmission electron microscopy (Figure 1c) showed vesicular structures in the size range typical for exosomes (50–130 nm). Immunoblot analysis was used to show the presence of Alix, a soluble protein characteristic for small EVs, as well as the absence of Calnexin, a marker protein found in endoplasmic reticulum membranes in our vesicle preparations. 

### 3.2. sEVs Transport MHC Class I Molecules from Melanoma Cells to APCs

**Figure 2:**
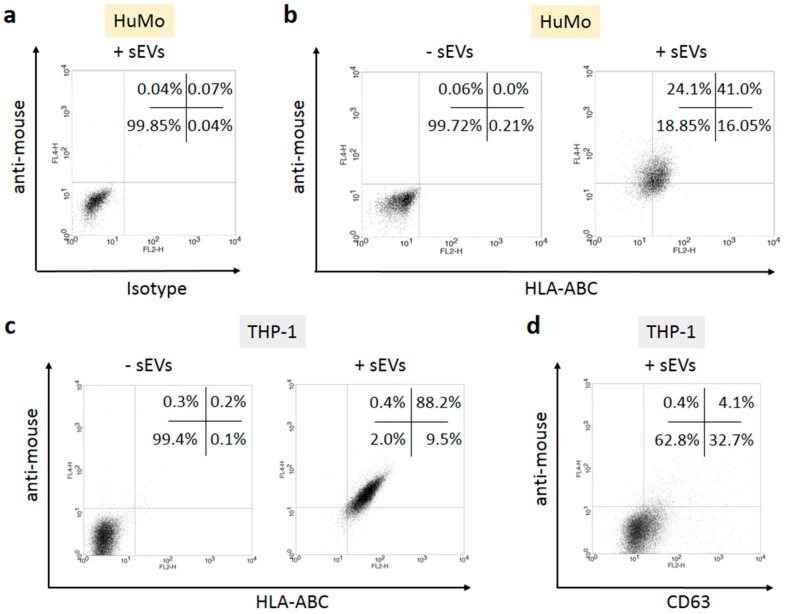
Melanoma cell-derived sEVs transfer MHC class I and class II molecules to the surface of APCs. The sEVs from A375 cells were incubated with PE-labelled isotype (**a**) or anti-HLA-ABC antibodies (**b**,**c**), purified by centrifugation, and incubated overnight with healthy donor monocytes (HuMo; **a**,**b**) or THP-1 cells (**c**,**d**). The next day, the cells were stained with APC-labelled secondary anti-mouse antibodies to demonstrate the appearance of the transferred MHC/antibody complexes on the surface of the recipient cells. A negative control following the same procedure but lacking sEVs is shown in the left part of (**b**,**c**). (**d**) Transfer of PE-labelled anti-CD63 antibodies through sEVs, followed by staining with anti-mouse antibodies. The percentages of events in the four quadrants are indicated. The results of one representative experiment out of three are shown.

To demonstrate the potential of sEVs to transfer melanoma-derived MHC molecules to APCs, sEVs were incubated with fluorescently labeled monoclonal mouse antibodies recognizing HLA-ABC (MHC class I) or CD63. Contaminating antibody complexes were eliminated by centrifugation, and unbound soluble antibodies were removed by pelleting the sEVs after diluting the antibody binding reaction about 100-fold with PBS. Primary human monocytes (Figure 2a,b) or THP-1 cells (Figure 2c,d) from a human monocytic cell line were incubated for 16 hours with the antibody-coated sEVs in cell culture. Flow cytometry measurements showed that the labeled antibodies bound to the MHC molecules were transferred to the target cells (Figure 2, shift along the *x*-axis). To demonstrate the specificity of the transfer, two kinds of negative controls were included, a sample containing sEVs mixed with isotype control antibodies (Figure 2a) and another sample containing HLA-ABC antibodies, but no sEVs. The isotype control confirmed that antibodies reached the target cells only when bound to specific antigens on the surface of sEVs. The negative control lacking sEVs (‘-sEVs’) showed that no detectable amount of free antibody was transferred by the applied procedure. To demonstrate that the sEV-transported MHC/antibody complexes appeared at the cell surface, the recipient cells were incubated with anti-mouse secondary antibodies (Figure 2, shift along the *y*-axis). These antibodies could bind only to the primary antibodies which appeared on the cell surface. The results demonstrated that the transported MHC class I complexes became exposed at the cell membrane. When an antibody to CD63 was transported by sEVs, the fluorescent label of the primary antibody was transferred to the cells, but the antibody did not appear at the cell surface as visualized by the low percentage (4.1%) of cells stained by the secondary anti-mouse antibody (Figure 2d).

### 3.3. sEV Mediated Modulation of Immune Receptor Surface Expression on APCs

**Figure 3:**
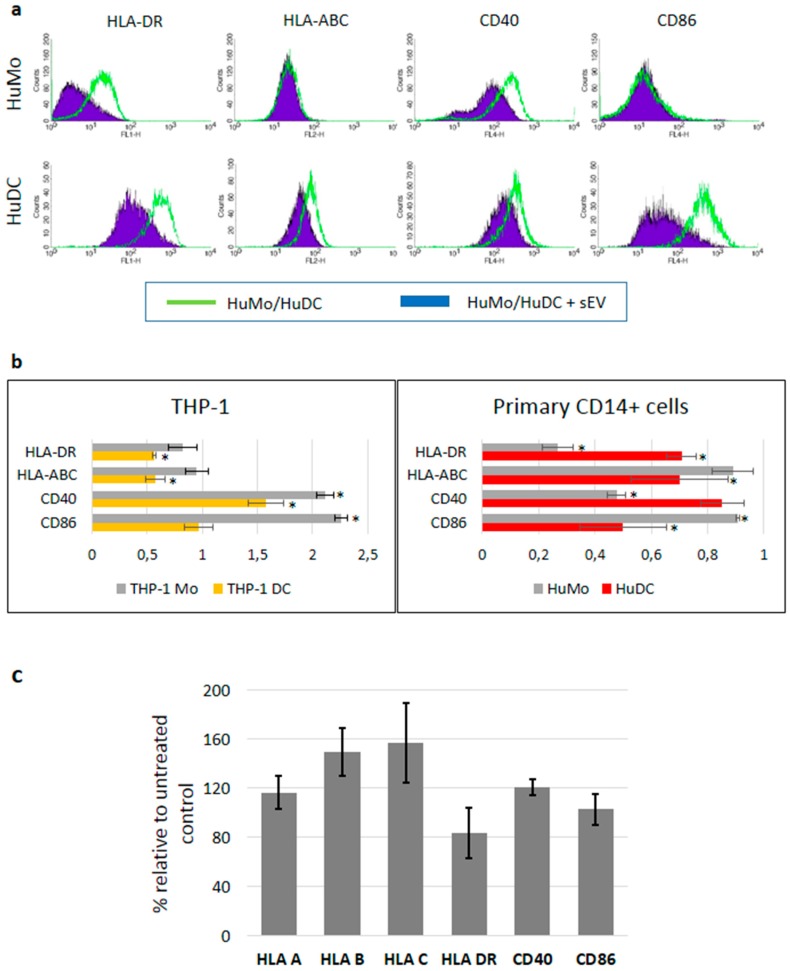
Melanoma cell-derived sEVs modulate the immune receptor expression on APCs. (**a**) Upper panel: The cell surface expression of MHC class I, II, CD40, and CD86 of CD14+ primary human monocytes (HuMo) was measured by flow cytometry (green lines). Overlaid are the histograms after exposure to A375 melanoma-derived sEVs for 48 hours (filled violet peaks; representative of n = 6). Lower panel: HuMo were differentiated into DCs (HuDC) and exposed to sEVs for 48 hours and the cell surface expression was analyzed by flow cytometry, as above (representative of n = 3). (**b**) The mean fluorescence intensities (MFI) of the surface expression of MHC class I, II, CD40, and CD86 after exposure to melanoma-derived sEVs was related to the MFI of unexposed cells. Both monocytes and DCs derived from either the THP-1 cell line (left diagram) or primary human CD14+ cells (right diagram) were used in the experiments. At least three independent experiments were conducted for each cell type and significant differences were marked by asterisks (Student’s t-test). (**c**) The mRNA expression levels in DC cells derived from the peripheral blood of healthy donors treated with A375-derived sEVs relative to untreated control cells. Mean values and standard deviations are shown from three independent experiments. The differences between samples after sEV exposure and untreated controls did not reach statistical significance (Student’s *t*-test).

To demonstrate the immunosuppressive capacity of melanoma-derived sEVs, human monocytes, and DCs derived thereof by in vitro culture were incubated with these sEVs. In preliminary experiments (not shown) an optimal sEV concentration of 20 µg/mL for the treatment of target cells (corresponding to approximately 1000–1250 vesicles/cell) was found to induce marked and consistent effects. Incubation of primary human monocytes (HuMo) with sEVs derived from A375 melanoma cells decreased the expression of co-receptor protein CD40 and HLA-DR, while the mean fluorescence intensity of HLA-ABC molecules and CD86 remained largely unchanged (Figure 3a, upper panel). In similar experiments using THP-1 cells, down-regulation was observed only for HLA-DR, while CD40 and CD86 cell surface expression was increased (Figure 3b). When CD14+ primary human monocytes were first differentiated into DCs (Figure 3a, lower panel), exposure to A375 cell-derived sEVs down-regulated the surface expression levels of MHC proteins class I and II, CD86 and CD40. DCs derived from THP-1 cells treated with A375-derived sEVs showed down-regulation of HLA molecules, while CD40 was up-regulated and CD86 stayed unchanged (Figure 3b). Using DCs derived from the peripheral blood of healthy donors, the expression of the investigated receptors was also assessed at the mRNA level (Figure 3c). Exposure to A375 melanoma sEVs reduced the HLA-DR mRNA level to about 80% of the untreated control, while class I MHC molecules and CD40 were slightly up-regulated (<1.5-fold). CD86 mRNA levels remained unchanged. 

### 3.4. sEVs Affect the Cytokine Expression of APCs

**Figure 4:**
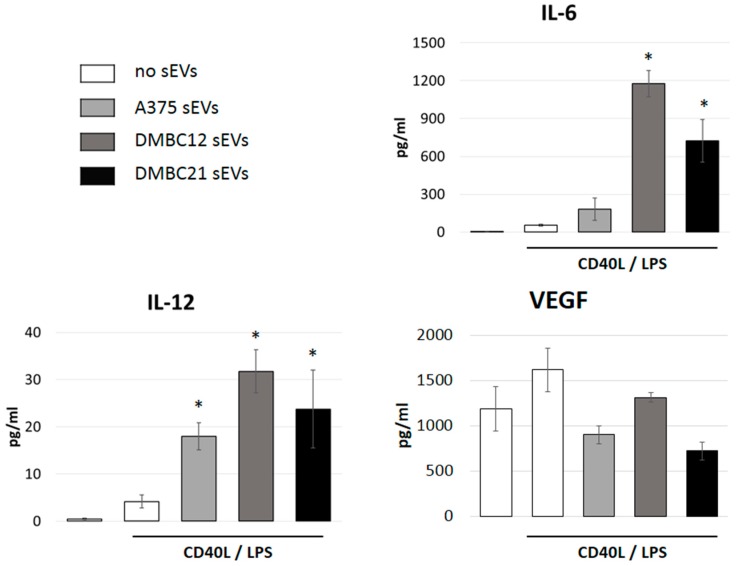
sEVs affect the cytokine secretion of APCs. THP-1 DC cells were stimulated to secrete cytokines by incubation with LPS and CD40L. The addition of melanoma cell-derived sEVs (A375, DMBC12 or DMBC21) increased the expression levels of IL-12 and IL-6, while secretion of VEGF was less affected. Mean values and standard deviations are shown from three independent experiments. Significant differences in the presence versus absence of sEVs are marked with asterisks.

THP-1 cells were differentiated into immature DCs and stimulated with CD40L and LPS [28]. The production of cytokines was assessed by cytometric bead assays using the cell culture supernatants. Concomitant incubation with sEVs that had been isolated from the A375 cell line, or patient-derived melanoma cells DMBC12 and DMBC21, increased the secretion of IL-12, as well as IL-6, while the levels of VEGF were slightly reduced (Figure 4).

### 3.5. sEVs Reduce T Cell Proliferation in Allogeneic Mixed Lymphocyte Reactions

**Figure 5:**
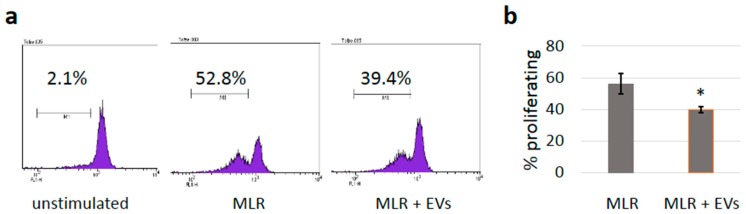
Mixed lymphocyte reaction (MLR). (**a**) Representative MLR experiment showing the induction of T cell proliferation by allogeneic PBMCs. PBMCs from one donor were labeled with CFSE to determine the proportion of cells undergoing cell division as each cell division reduces the mean fluorescence intensity by half. The analysis was restricted to T cells by gating on CD3-positive cells. (**b**) Summary of three independent experiments showing the percentage of proliferating cells in the presence or absence of sEVs (mean values +/- SD); a significant difference was found with *p* = 0.045 (Student’s *t*-test).

To investigate whether the reduced expression of immune receptors would also have functional consequences, mixed lymphocyte reactions (MLRs) were performed. Co-culturing the PBMCs isolated from healthy donors resulted in the stimulation of T cell proliferation based on allogeneic reactivity. Labeling the cells of one donor with CFSE allowed for determining the proportion of cells undergoing cell division, as each cell division halves the mean fluorescence intensity. By gating on CD3-positive cells, the analysis was restricted to T cells. As shown in Figure 5, the presence of A375 sEVs reduced the percentage of proliferating T cells in MLRs slightly, but significantly (*p* = 0.045).

### 3.6. sEVs Transfer TGF-β with Immunosuppressive Activity

**Figure 6:**
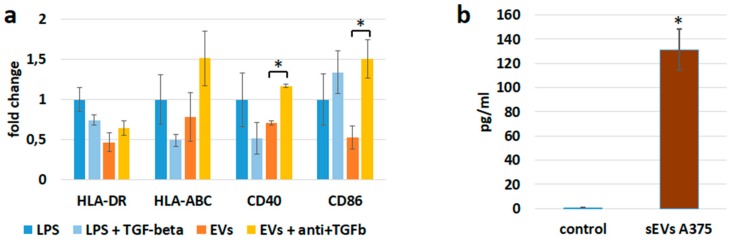
The down-regulation of receptor molecules on APCs could be partially caused by TGF-β. (**a**) Incubation of PBMCs differentiated into DCs with 20 pg/mL recombinant TGF-β reduced the expression of CD40 and MHC class I and II molecules, measured by flow cytometry. Reduced expression of all four antigens after incubation with sEVs derived from A375 melanoma cells was reversed in the presence of anti-TGFβ antibodies (1 µg/mL). The expression of MHC molecules, CD40 and CD80, was evaluated by flow cytometry. The mean fluorescence intensities (MFI) relative to untreated control cells are shown. Significant differences between samples treated with sEVs or with sEVs and anti-TGF-β antibodies are marked by asterisks. (**b**) TGF-β was measured by a cytometric bead assay in A375 cell-derived sEVs. A sample without sEVs served as a negative control.

sEVs can transfer immunosuppressive cytokines, such as TGF-β. Control experiments using recombinant TGF-β showed that this cytokine was able to down-regulate the expression of MHC class I and class II and CD40 molecules (Figure 6a, compare the first two columns for each antigen). Therefore, we measured TGF-β directly in sEV preparations (Figure 6b). TGF-β could be identified in quantities sufficient to induce the observed effects on APCs. Recombinant TGF-β was used in a concentration of 20 pg/mL. In experiments assessing the inhibitory effect on immune receptor expression, the sEVs were used at a concentration of 20 µg protein per mL of medium. Consequently, about 1 pg TGF-β per µg protein should be sufficient to obtain similar effects as with the recombinant cytokine. The amount of TGF-β measured in the sEV preparations was in the range of 10–15 pg/µg protein (Figure 6b). TGF-β neutralizing antibodies, along with sEVs, reduced the sEV-mediated down-regulation of HLA-ABC, HLA-DR, CD40, and CD86 (Figure 6a, third and fourth columns). These results suggest that TGF-β participated in the down-regulation of immune receptors during the incubation with sEVs.

## 4. Discussion

The suppression of anti-cancer immune responses by tumors is central to their survival. Cancer-induced immunosuppression is antigen-specific rather than systemic and we provide evidence that melanoma-derived sEVs are able to induce TAA-specific tolerance. sEVs have to meet two requirements to function as mediators of TAA-specific immune suppression, as follows: They have to transfer MHC molecules from cancer cells to APCs and, simultaneously, they have to promote an immunosuppressive phenotype. We demonstrate that MHC class I receptor proteins are transported to APCs by sEVs where they appear at the cell surface (Figure 2). In contrast, CD63 carried by sEVs did not reach the target cell surface (Figure 2d), suggesting that the intracellular distribution of vesicular proteins is regulated and only specific proteins become exposed at the cell surface. 

The second requirement, the immunosuppressive activity of melanoma-derived extracellular vesicles, was demonstrated by down-regulation of MHC molecules and co-stimulatory receptors on monocytes and DCs (Figure 3), induced production of IL-6 (Figure 4), and reduced T cell proliferation in MLRs (Figure 5). The immunosuppressive activity of tumor-derived EVs has been well documented earlier. Exosomes triggered apoptosis of CD8 T cells, impaired cytotoxic T cell functions, and induced conversion of naïve T cells into Treg cells [31]. The latter can occur either directly through transported cytokines and metabolic enzymes [32,33] or indirectly involving MDSCs [32]. TGF-β and PgE2 are able to induce MDSCs in mice [34] and humans [35] and both factors were found to be transported by exosomes [34]. In humans, the additional contribution of Hsp70 and miRNAs was demonstrated [36,37,38]. Exosomes were implicated in antigen-specific tolerance, as exosomes isolated from the blood of antigen-fed mice or tumor-bearing mice were able to suppress delayed-type hypersensitivity (DTH) in an antigen-specific manner [39,40,41]. When isolated from the blood of tumor-bearing mice, exosomes suppressed the inflammation caused by a defined tumor antigen. The presence of MHC class II and FasL on these exosomes was of crucial importance for their suppressive activity [40]. 

In our experimental setting, the treatment with cancer-derived sEVs changed the receptor expression on APCs in different ways. First, the sEVs are allogeneic to the APCs, thus they exert a non-specific stimulation [42]. APC activation usually includes up-regulation of MHC molecules and co-stimulatory receptors, similar to incubation with LPS. Indeed, an elevated expression of CD40 and CD86 was observed in THP-1 cells exposed to melanoma-derived sEVs (Figure 3). In line with these results, Bretz et al. demonstrated that the stimulation of THP-1 cells by exosomes of cancerous and non-cancerous origin occurred through a TLR (toll-like receptor) dependent mechanism [42]. Furthermore, tumor-derived sEVs have been shown to activate primary monocytes [43]. We used sEVs isolated from a well-established melanoma cell line (A375), as well as from patient-derived primary melanoma cell lines. A second mechanism changing the expression level of MHC receptors is the direct transfer of HLA-ABC by sEVs to the surface of APCs. Third, vesicle-transported mediators, including cytokines and miRNAs, can change the expression of immune receptors. We demonstrated that TGF-β, one of the most powerful immunosuppressive cytokines, was present in sEV preparations in sufficient amounts to cause the observed reduction of MHC and CD40 expression (Figure 6). Exosomes carry a latent form of TGF-β with full biological activity in their membrane [44]. We confirmed the potential of TGF-β to mediate the down-regulation of immune receptors and show that the presence of anti-TGF-β neutralizing antibodies reversed the sEV-mediated down-regulation of MHC molecules CD40 and CD86 (Figure 6). The expression levels of HLA-ABC, CD40, and CD86 in the presence of anti-TGF-β neutralizing antibodies even exceeded the one of control cells, indicating that sEVs by themselves exert immune stimulatory effects that are outperformed by TGF-β mediated suppression. TGF-β transported by exosomes was recently shown to induce Treg cells in gastric cancer [45]. Furthermore, depletion of TGF-β from leukemia cell-derived exosomes increased the immune stimulatory potential of these vesicles, inducing potent anti-leukemic immunity [46]. We suggest that inhibition of DC maturation visualized by immune receptor down-regulation contributed to the observed immunosuppressive effects. Corroborating our findings, impairment of DC differentiation upon exposure to lung carcinoma-derived exosomes was recently demonstrated in mice [47].

While the protein expression was markedly modulated by sEV exposure, corresponding changes in the mRNA levels for HLA class I and II, CD40, and CD86 were not detected. The modulation of immune receptors seems to occur at a post-transcriptional level and might be caused by miRNAs contained in sEVs. MiRNAs inhibit translation of specific target mRNAs by binding to complementary sequences. MiRNAs from exosomes released by melanoma cells have been shown to play multiple roles in promoting tumor development [48]. In our microarray analysis, published recently, 386 common miRNAs were detected in sEVs derived from four primary melanoma cell lines [49]. 

Interestingly, contact with sEVs also led to the induction of cytokine secretion (Figure 4). We observed a strong up-regulation of pro-inflammatory IL-6 secretion by sEV-exposed APCs. This interleukin seems to play a major role in melanoma pathogenesis. Through an autocrine loop, including the activation of STAT3, IL-6 signaling contributes to survival and proliferation of melanoma cells, increases the metastatic potential, and facilitates immune evasion [50]. More specifically, IL-6 mediated STAT3 activation in DCs was shown to down-regulate MHC class II molecules, resulting in the inhibition of the functional maturation of DCs [51]. IL-6 promotes MDSC generation [52] and has been shown to impair T helper 1 cell differentiation [53]. In contrast to Il-6, which was virtually absent in control cell cultures, VEGF was secreted in relatively high amounts, also in the absence of sEVs. The incubation with sEVs left VEGF levels mostly unchanged. VEGF was shown to correlate with MDSC infiltration into tumor tissue [54]. 

In line with a predominant immunosuppressive function of melanoma-derived EVs, reduced T cell proliferation in MLRs was also measured (Figure 5). Some of the observed effects were relatively mild, raising the question about their relevance in vivo. However, in our experiments, sEVs were applied only once while, in tumor-bearing organisms, a constant flow of sEVs may cause accumulative effects. Furthermore, in the patients, the sEVs produced by cancer cells are autologous and exert no allogeneic stimulation. We tried to avoid extreme sEV concentrations, which might raise non-specific effects. However, exploration of dose effects could further improve our understanding and, for the future, experiments are planned to demonstrate that the immunosuppressive effects observed in our study are highly relevant to the disease.

## 5. Conclusions

In summary, exposure to melanoma-derived sEVs shifts APCs towards an immunosuppressive phenotype, with down-regulated expression of immune receptors that are required for the optimal stimulation of a cytotoxic T cell response. Immunosuppression as cancer-associated deregulation involves many mechanisms and the antigen-specific immunosuppression through sEVs represents one plausible strategy.

## Supplementary Materials

The following is available online at https://www.mdpi.com/2073-4409/8/7/665/s1, Table S1: Primers used for amplification of mRNAs.

## References

[1] Jazirehi A.R., Lim A., Dinh T. (2016). PD-1 inhibition and treatment of advanced melanoma-role of pembrolizumab. Am. J. Cancer Res..

[2] Weiss S.A., Wolchok J.D., Sznol M. (2019). Immunotherapy of Melanoma: Facts and Hopes. Clin. Cancer Res..

[3] Chen D.S., Mellman I. (2017). Elements of cancer immunity and the cancer-immune set point. Nature.

[4] Escors D. (2014). Tumour immunogenicity, antigen presentation and immunological barriers in cancer immunotherapy. New J. Sci..

[5] Melief C.J. (2003). Regulation of cytotoxic T lymphocyte responses by dendritic cells: peaceful coexistence of cross-priming and direct priming?. Eur. J. Immunol..

[6] Nel A.E. (2002). T-cell activation through the antigen receptor. Part 1: signaling components, signaling pathways, and signal integration at the T-cell antigen receptor synapse. J. Allergy Clin. Immunol..

[7] Mescher M.F., Curtsinger J.M., Agarwal P., Casey K.A., Gerner M., Hammerbeck C.D., Popescu F., Xiao Z. (2006). Signals required for programming effector and memory development by CD8+ T cells. Immunol. Rev..

[8] Zheng Y., Manzotti C.N., Liu M., Burke F., Mead K.I., Sansom D.M. (2004). CD86 and CD80 differentially modulate the suppressive function of human regulatory T cells. J. Immunol..

[9] Chen L., Hasni M.S., Jondal M., Yakimchuk K. (2017). Modification of anti-tumor immunity by tolerogenic dendritic cells. Autoimmunity.

[10] Kuwana M. (2002). Induction of anergic and regulatory T cells by plasmacytoid dendritic cells and other dendritic cell subsets. Hum. Immunol..

[11] Wang L., Pino-Lagos K., de Vries V.C., Guleria I., Sayegh M.H., Noelle R.J. (2008). Programmed death 1 ligand signaling regulates the generation of adaptive Foxp3+CD4+ regulatory T cells. Proc. Natl. Acad. Sci. USA.

[12] Walker L.S., Sansom D.M. (2011). The emerging role of CTLA4 as a cell-extrinsic regulator of T cell responses. Nat. Rev. Immunol..

[13] Fritz J.M., Lenardo M.J. (2019). Development of immune checkpoint therapy for cancer. J. Exp. Med..

[14] Rabinovich G.A., Gabrilovich D., Sotomayor E.M. (2007). Immunosuppressive strategies that are mediated by tumor cells. Annu. Rev. Immunol..

[15] Mittal S.K., Roche P.A. (2015). Suppression of antigen presentation by IL-10. Curr. Opin. Immunol..

[16] Sheng J., Chen W., Zhu H.J. (2015). The immune suppressive function of transforming growth factor-β (TGF-β) in human diseases. Growth Factors.

[17] Voron T., Marcheteau E., Pernot S., Colussi O., Tartour E., Taieb J., Terme M. (2014). Control of the immune response by pro-angiogenic factors. Front. Oncol..

[18] Zhou G., Drake C.G., Levitsky H.I. (2006). Amplification of tumor-specific regulatory T cells following therapeutic cancer vaccines. Blood.

[19] Li X., Wang Y., Wang Q., Liu Y., Bao W., Wu S. (2018). Exosomes in cancer: Small transporters with big functions. Cancer Lett..

[20] Kaiser J. (2016). Malignant messengers. Science.

[21] Gurunathan S., Kang M.H., Jeyaraj M., Qasim M., Kim J.H. (2019). Review of the Isolation, Characterization, Biological Function, and Multifarious Therapeutic Approaches of Exosomes. Cells.

[22] Czernek L., Düchler M. (2017). Functions of Cancer-Derived Extracellular Vesicles in Immunosuppression. Arch. Immunol. Ther. Exp. (Warsz)..

[23] McConnell M.J. (2018). Extracellular vesicles and immune modulation. Immunol. Cell Biol..

[24] Anel A., Gallego-Lleyda A., Miguel D., Naval J., Martínez-Lostao L. (2019). Role of Exosomes in the Regulation of T-Cell Mediated Immune Responses and in Autoimmune Disease. Cells.

[25] Whiteside T.L. (2013). Immune modulation of T-cell and NK (natural killer) cell activities by TEXs (tumour-derived exosomes). Biochem. Soc. Trans..

[26] Norbury C.C. (2016). Defining cross-presentation for a wider audience. Curr. Opin. Immunol..

[27] Sztiller-Sikorska M., Hartman M.L., Talar B., Jakubowska J., Zalesna I., Czyz M. (2015). Phenotypic diversity of patient-derived melanoma populations in stem cell medium. Lab. Investig..

[28] Czernek L., Chworos A., Düchler M. (2015). The Uptake of Extracellular Vesicles is Affected by the Differentiation Status of Myeloid Cells. Scand. J. Immunol..

[29] Parisse P., Rago I., Ulloa Severino L., Perissinotto F., Ambrosetti E., Paoletti P., Ricci M., Beltrami A.P., Cesselli D., Casalis L. (2017). Atomic force microscopy analysis of extracellular vesicles. Eur. Biophys J..

[30] Manek R., Moghieb A., Yang Z., Kumar D., Kobessiy F., Sarkis G.A., Raghavan V., Wang K.K.W. (2018). Protein Biomarkers and Neuroproteomics Characterization of Microvesicles/Exosomes from Human Cerebrospinal Fluid Following Traumatic Brain Injury. Mol. Neurobiol..

[31] Wieckowski E.U., Visus C., Szajnik M., Szczepanski M.J., Storkus W.J., Whiteside T.L. (2009). Tumor-derived microvesicles promote regulatory T cell expansion and induce apoptosis in tumor-reactive activated CD8+ T lymphocytes. J. Immunol..

[32] Sharma S., Yang S.C., Zhu L., Reckamp K., Gardner B., Baratelli F., Huang M., Batra R.K., Dubinett S.M. (2005). Tumor cyclooxygenase-2/prostaglandin E2-dependent promotion of FOXP3 expression and CD4+ CD25+ T regulatory cell activities in lung cancer. Cancer Res..

[33] Chen W., Jin W., Hardegen N., Lei K.J., Li L., Marinos N., McGrady G., Wahl S.M. (2003). Conversion of peripheral CD4+ CD25− naive T cells to CD4+ CD25+ regulatory T cells by TGF-β induction of transcription factor Foxp3. J. Exp. Med..

[34] Ghiringhelli F., Puig P.E., Roux S., Parcellier A., Schmitt E., Solary E., Kroemer G., Martin F., Chauffert B., Zitvogel L. (2005). Tumor cells convert immature myeloid dendritic cells into TGF-β secreting cells inducing CD4+CD25+ regulatory T cell proliferation. J. Exp. Med..

[35] Xiang X., Poliakov A., Liu C., Liu Y., Deng Z.B., Wang J., Cheng Z., Shah S.V., Wang G.J., Zhang L. (2009). Induction of myeloid-derived suppressor cells by tumor exosomes. Int. J. Cancer.

[36] Montecalvo A., Larregina A.T., Shufesky W.J., Stolz D.B., Sullivan M.L.G., Karlsson J.M., Baty C.J., Gibson G.A., Erdos G., Wang Z. (2012). Mechanism of transfer of functional microRNAs between mouse dendritic cells via exosomes. Blood.

[37] Chalmin F., Ladoire S., Mignot G., Vincent J., Bruchard M., Remy-Martin J.P., Boireau W., Rouleau A., Simon B., Lanneau D. (2010). Membrane-associated Hsp72 from tumor-derived exosomes mediates STAT3-dependent immunosuppressive function of mouse and human myeloid-derived suppressor cells. J. Clin. Investig..

[38] Fernández-Messina L., Gutiérrez-Vázquez C., Rivas-García E., Sánchez-Madrid F., de la Fuente H. (2015). Immunomodulatory role of microRNAs transferred by extracellular vesicles. Biol. Cell..

[39] Greening D.W., Gopal S.K., Xu R., Simpson R.J., Chen W. (2015). Exosomes and their roles in immune regulation and cancer. Semin. Cell Dev. Biol..

[40] Yang C., Ruffner M.A., Kim S.H., Robbins P.D. (2012). Plasma-derived MHC class II+ exosomes from tumor-bearing mice suppress tumor antigen-specific immune responses. Eur. J. Immunol..

[41] Kim S.H., Bianco N.R., Shufesky W.J., Morelli A.E., Robbins P.D. (2007). MHC class II+ exosomes in plasma suppress inflammation in an antigen-specific and Fas ligand/Fas-dependent manner. J. Immunol..

[42] Bretz N.P., Ridinger J., Rupp A.K., Rimbach K., Keller S., Rupp C., Marmé F., Umansky L., Umansky V., Eigenbrod T. (2013). Body fluid exosomes promote secretion of inflammatory cytokines in monocytic cells via Toll-like receptor signaling. J. Biol. Chem..

[43] Gärtner K., Battke C., Dünzkofer J., Hüls C., von Neubeck B., Kellner M.K., Fiestas E., Fackler S., Lang S., Zeidler R. (2018). Tumor-derived extracellular vesicles activate primary monocytes. Cancer Med..

[44] Webber J., Steadman R., Mason M.D., Tabi Z., Clayton A. (2010). Cancer exosomes trigger fibroblast to myofibroblast differentiation. Cancer Res..

[45] Yen E.Y., Miaw S.C., Yu J.S., Lai I.R. (2017). Exosomal TGF-β1 is correlated with lymphatic metastasis of gastric cancers. Am. J. Cancer Res..

[46] Huang F., Wan J., Hao S., Deng X., Chen L., Ma L. (2017). TGF-β1-silenced leukemia cell-derived exosomes target dendritic cells to induce potent anti-leukemic immunity in a mouse model. Cancer Immunol. Immunother..

[47] Ning Y., Shen K., Wu Q., Sun X., Bai Y., Xie Y., Pan J., Qi C. (2018). Tumor exosomes block dendritic cells maturation to decrease the T cell immune response. Immunol. Lett..

[48] Gajos-Michniewicz A., Düchler M., Czyz M. (2014). MiRNA in melanoma-derived exosomes. Cancer Lett..

[49] Wozniak M., Peczek L., Czernek L., Düchler M. (2017). Analysis of the miRNA Profiles of Melanoma Exosomes Derived Under Normoxic and Hypoxic Culture Conditions. Anticancer Res..

[50] Kortylewski M., Jove R., Yu H. (2005). Targeting STAT3 affects melanoma on multiple fronts. Cancer Metastasis Rev..

[51] Kitamura H., Ohno Y., Toyoshima Y., Ohtake J., Homma S., Kawamura H., Takahashi N., Taketomi A. (2017). Interleukin-6/STAT3 signaling as a promising target to improve the efficacy of cancer immunotherapy. Cancer Sci..

[52] Lee B.R., Kwon B.E., Hong E.H., Shim A., Song J.H., Kim H.M., Chang S.Y., Kim Y.J., Kweon M.N., Youn J.I. (2016). Interleukin-10 attenuates tumour growth by inhibiting interleukin-6/signal transducer and activator of transcription 3 signalling in myeloid-derived suppressor cells. Cancer Lett..

[53] Tsukamoto H., Nishikata R., Senju S., Nishimura Y. (2013). Myeloid-derived suppressor cells attenuate TH1 development through IL-6 production to promote tumor progression. Cancer Immunol. Res..

[54] Horikawa N., Abiko K., Matsumura N., Hamanishi J., Baba T., Yamaguchi K., Yoshioka Y., Koshiyama M., Konishi I. (2017). Expression of Vascular Endothelial Growth Factor in Ovarian Cancer Inhibits Tumor Immunity through the Accumulation of Myeloid-Derived Suppressor Cells. Clin. Cancer Res..

