# Supplementary material for: Melanoma-Derived Extracellular Vesicles Bear the Potential for the Induction of Antigen-Specific Tolerance
Source: Cells. 2019 Jul 2;8(7):665. doi: 10.3390/cells8070665 (PMC6679195; doi:10.3390/cells8070665)
Supplement: Supplementary file 1 [file cells-08-00665-s001.pdf]

Supplementary file

Supplementary Table 1. Primers used for amplification of mRNAs (forward – F and reverse - R)

Amplified gene	NCBI Reference Sequence	Primer sequence	Amplicon size (bp)
CD86	NM_175862.4	F: 5’ – GAG GGG TTT TGG TGA TAC CC – 3’<br>R: 5’ – TTA TCA AGG TGA TGG CTC TCC – 3’	83
CD80	NM_005191.3	F: 5’ – AAC CAA CTA TCC TGT CTT TAA GTG C – 3’<br>R: 5’ – GCA GCA TAT CAC AAA AAT TCC AT – 3’	110
CD40	NM_001250.5	F: 5’ – ACC TCG CTA TGG TTC GTC TG – 3’<br>R: 5’ – CAG TGG GTG GTT CTG GAT G – 3’	81
HLA-A	NM_002116.7	F: 5’ – TTG AGA GCC TAC CTG GAT GG – 3’<br>R: 5’ – TGG TGG GTC ATA TGT GTC TTG – 3’	110
HLA-B	NM_005514.7	F: 5’ – CCT ACC TGG AGG GCG AGT – 3’<br>R: 5’ – GGT GGG TCA CGT GTG TCT TT – 3’	102
HLA-C	NM_002117.5	F: 5’ – ATA CCT GGA GAA CGG GAA GG – 3’<br>R: 5’ – TGG CCT CAT GGT CAG AGA G – 3’	89
HLA-DR alpha	NM_019111.4	F: 5’ – CAA GGG ATT GCG CAA AAG – 3’<br>R: 5’ – AAG CAG AAG TTT CTT CAG TGA TCT T – 3’	106
